# Mesenchymal and Proneural Subtypes of Glioblastoma Disclose Branching Based on GSC Associated Signature

**DOI:** 10.3390/ijms22094964

**Published:** 2021-05-07

**Authors:** Giedrius Steponaitis, Arimantas Tamasauskas

**Affiliations:** Laboratory of Molecular Neurooncology, Neuroscience Institute, Lithuanian University of Health Sciences, LT-50161 Kaunas, Lithuania; arimantas.tamasauskas@kaunoklinikos.lt

**Keywords:** glioblastoma, glioma stem cells, subtyping, biomarkers, mesenchymal, proneural

## Abstract

Glioblastomas (GBM)—the most common, therapy-resistant, and lethal tumors driven by populations of glioma stem cells (GSCs) are still on the list of the most complicated pathologies. Thus, deeper understanding and characterization of GSCs is indispensable to find suitable targets and develop more effective therapies. In the present study, we applied native glioblastoma cells and GSCs sequencing, screened for GSC-specific targets and checked if the signature is related to GBM patient pathological, clinical data as well as molecular subtypes applying TCGA cohort. Data analysis revealed that tumors of proneural and mesenchymal subtypes are branching in separate clusters based on screened gene expression. Samples of the same subtype revealed significantly different patient survival prognosis as well as recurrence chance between the clusters. Recently, different subpopulations of mesenchymal GSC demonstrating different properties were shown, which indicates possible internal heterogeneity of GBM subtypes as well. Current findings also revealed branching of molecular GBM subtypes that were significantly linked to patient outcome and that might be decided by distinct GSC subpopulations.

## 1. Introduction

The main feature of glioblastoma (GBM) tumors is their intrinsic resistance to currently applied therapies that leads to extremely poor clinical outcomes and survival that rarely exceeds 15 months after the diagnosis [1]. Even after maximal gross-total resection at surgery of well-demarcated tumors and subsequent combined irradiation and chemotherapy, the vast majority of tumors are recurring and resulting in a dismal outcome, since recurred GBMs are resistant to therapy. The main factor previously indicated to be at least partially responsible for poor patient outcome was inter- and intra-heterogeneity of GBMs which does not allow for proper treatment [2]. Genome-wide transcriptome analysis led to classify GBM into four more homogenous subtypes: mesenchymal (MES), classical (CL), proneural (PN) and neural (NE) based on bulk tumor transcription profile [3], nevertheless the clinical relevance of subtyping is still debatable [4,5]. Over the years, substantial evidence has confirmed the existence of glioma initiating/propagating or cancer stem-like cells within GBMs [6]. The relapse, resistance to treatment and tumor maintenance was demonstrated to be driven by a small subpopulation of tumorigenic cells displaying stem-like properties—Glioma stem cells (GSC) [6]. GSC has similar properties as stem cells, and are able to sustain self-renewal, persist proliferation, have an ability to generate progeny of multiple lineages and initiate tumor upon secondary transplantation [7]. Thus, the properties of GSC assure the heterogeneity of glioblastomas. Besides heterogeneity of GBM, it was shown that at least two mutually distinct subtypes of glioma stem cells—Proneural and Mesenchymal can be found in tumors [8,9,10], that shows distinct rates of proliferation and sensitivity to therapy [11]. Similar to GBM subtypes (Verhaak et al., 2010) GSC subtypes were identified based on transcriptome screening and since type I and type II GSC signatures were associated with PN and MES GBM subtypes, GSC were called accordingly [9]. Besides molecular differences MES GSCs showed higher rates of proliferation in vitro and vivo when mice that received MES GSCs developed brain tumors much faster than PN GSCs received mice [11], MES GSCs are more angiogenic [12], invasive and resistant to radiation than PN GSCs [8]. Moreover, it was found that primary PN GBM after treatment often relapse as tumors having MES-like markers and become resistant to treatment [8,13]. Such transition is explained by two possible occurrences: first by PN-MES transition, where PN GSCs are triggered to switch to an MES phenotype upon treatment; and second by inherent tumor heterogeneity, where small numbers of treatment-resistant MES GSCs already are present in primary PN tumors which prosper and form a mass of recurring tumors after treatment [13,14]. Therefore, accurate identification of GSC and even different types of GSC in high-grade gliomas must be the upcoming task in order to eventually provide significant and personalized therapeutic strategies instead of applying a standard cure to all patients with GBM.

The GSC impact for glioblastoma progression is indisputable, nevertheless, we still underestimate GSC signature significance when measuring tumor characteristics. In present study we compiled GSC specific 30-gene signature to estimate patient clinical and pathological characteristics in prospect of GBM subtypes.

## 2. Results

### 2.1. Differentially Expressed Genes

**Figure 1:**
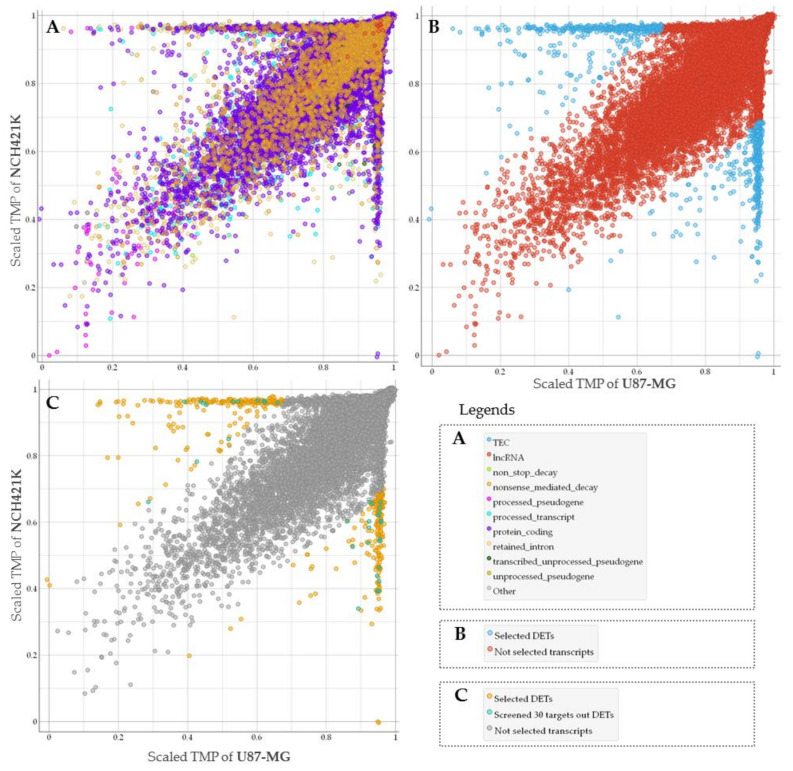
(**A**) The general view of all types of sequenced transcripts plotted based on NCH421K and U87-MG level (TMP—transcripts per million) distribution. TPM values scaled to interval from 0 to 1. (**B**) Screened molecules (represented in blue dots) according to differentially expressed transcripts (DETs) analysis. (**C**) Only protein-coding transcripts showed. Transcripts represented in turquoise dots were chosen for classifier based on feature selection model, yellow dots represent selected differentially expressed protein coding transcripts.

To assess GSC specific signal applicability for more accurate GBM patient survival prognostication in subtype groups we performed RNA sequencing of glioma stem cell line—NCH421K and glioblastoma cell line U87-MG. Nanopore sequencing in total resulted in 61,560 sequenced transcripts, of which 38,891 were common between U87-MG and NCH421K cells. Differential expression analysis using log2Fold Change as a scoring method (*p* < 0.05) was applied to select the most differentially expressed reads between cell lines what in turn yielded 859 transcripts, out of which 455 were upregulated in GSC while 404 transcripts were downregulated as compared to U87-MG cells. The was majority—382 were protein-coding, processed transcripts accounted for 107, 86 were nonsense-mediated decay reads, 270 retained intron, and 10 processed and unprocessed pseudogenes transcripts, Figure 1A,B.

### 2.2. Selection of GSC Specific Core Genes

Next, we used TCGA data of GBM mRNA expression to examine if screened GSC characteristic signature can be informative to evaluating patient clinical data. For this purpose, we selected protein-coding transcripts and applied a narrowing down analysis by feature selection model using univariate regression method to rank genes and select those, which demonstrated the strongest association with GBM patient survival. Finally, 30 of the most informative genes were selected, Figure 1C. Protein coding transcripts were selected since the vast majority of GBM expression targets analyzed by microarray at TCGA are protein-coding [15].

### 2.3. GSC Specific Genes and GBM Subtypes

**Figure 2:**
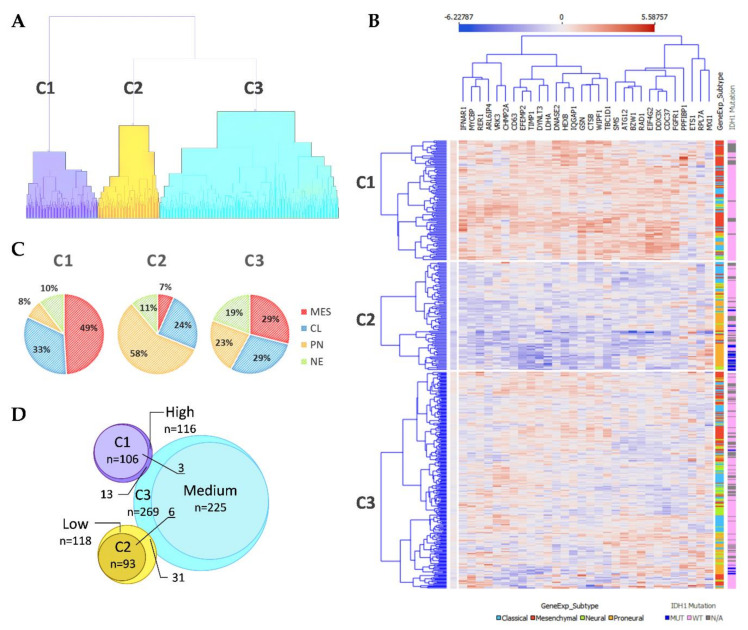
(**A**) Unsupervised hierarchal clustering of screened GSC signature genes applying TCGA GBM specimens generated three clusters. C1–3—Cluster 1–3. (**B**) Heatmap of TCGA GBM specimens using 30 GSC specific genes. Samples grouped according to cluster dependency. Penultimate column indicates GBM subtype: light blue color—Classical; red color—Mesenchymal; green color—Neural; orange color—Proneural. The last column indicates *IDH1* mutation status: dark blue color—Mutant case; pink—Wild type case; grey color—no data available. (**C**) GBM subtypes composition at the clusters. (**D**) Venn diagram of Clusters and average expression of selected 30 genes groups (grouped in low, medium, and high expression groups according first (Q1) and third (Q3) quantiles) specimens overlapping.

**Figure 3:**
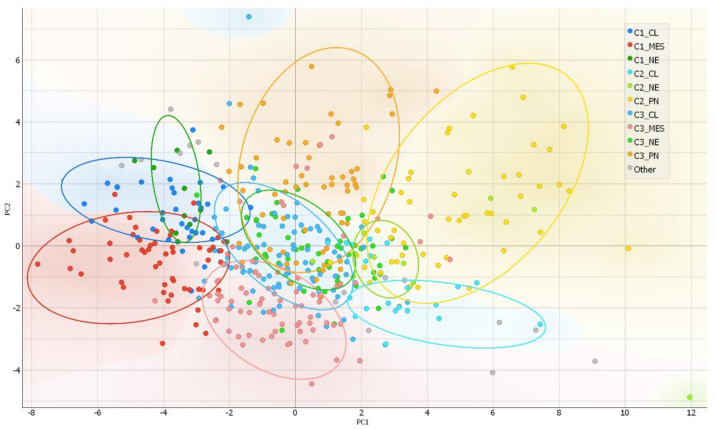
PCA as a complexity reduction model was applied to compress data in to lower-dimensional feature subspace for data visualization. Two-dimensional scatterplot of samples distribution based on to first two PCA (representing 50.8% of data) conducted from 30-gene GSC signature.

After the ranking and reduction of the target genes, we performed unsupervised hierarchal clustering analysis which revealed TCGA GBM patient grouping into three main clusters, Figure 2A. We also calculated an average expression of selected 30 genes and grouped samples into low, medium, and high expression groups according to first (Q1) and third (Q3) quantiles. Hierarchal clustering revealed very similar matching of the samples, where cluster 1 (C1) mainly consisted of high expression group samples, cluster 2 (C2) was consisted of low expression group cases and cluster 3 (C3) mainly consisted of medium expression group samples, Figure 2D. Then we checked for GBM subtype distribution in clusters and noticed that the dominant subtype in C1 was mesenchymal (49%) while proneural subtype was dominant (58%) in Cluster 3 consisted of relatively equal number of subtypes, Figure 2C. Data showed inherent distance between MES and PN subtypes, since the PN part accounted for only 8% in C1, while the MES part in C2 accounted for only 7% and even in C3 MES and PN were located at distal sides of separate subclusters of C3, see Figure 2B. Chi-square test confirmed significant subtypes frequency distribution between clusters, Pearson χ^2^ = 84.46, df = 6, *p* < 0.From the heatmap analysis can be seen that the vast majority of screened genes were upregulated in C1, while in C2 the same genes were downregulated. Cluster 3 showed intermediate levels, nevertheless, the profile slightly differed depending on the sample, Figure 2B. To reveal data points distributions and distances between clusters and subtypes we compressed data in to lower-dimensional feature subspace by performing principal component analysis (PCA) of 30-genes expression data and used first two components (representing 50.8% of data) for visualization, Figure 3. The generated scatterplot confirms that MES and PN samples were the most distal and do not overlap, while CL and NE remained in between MES and PN, Figure 3.

*IDH1* mutations, that are exceptionally intrinsic to PN subtype distributed in C2 and C3, Figure 2B. Pearson Chi-square test showed a significantly different distribution of mutant cases between all clusters of proneural samples (χ^2^ = 8.53, df = 2, *p* = 0.014, *n* = 91); as well as comparing only C2 and C3 (χ^2^ = 5.53, df = 1, *p* = 0.019, *n* = 84). Similarly, frequency distribution analysis revealed different proportions of *IDH1* mutation cases between cluster when comparing the entire cohort (χ^2^ = 40.62, df = 2, *p* < 0.001, *n* = 468). Pearson Chi-square test revealed that G-CIMP [16], as well as methylation groups (M1-M6) defined by Brennan et al., 2013 [17], was significantly associated with clusters, (χ^2^ = 67.3, df = 2, *p* < 0.001, *n* = 459) and (χ^2^ = 20, df = 8, *p* = 0.01, *n* = 422), respectively. *MGMT* promoter methylation was not associated with clusters, Table 1.

The apparent separation of subtypes between cluster groups were characteristic for all subtypes. Obvious subtypes partition into different clusters let us assume that tumors of the same subtype but distinct clusters might be related to different course of the disease, hence the clinical outcome of patients and disease biology. Therefore, we further checked for supposed connections.

### 2.4. Patient Clinical and Pathological Data Association to Clusters

**Figure 4:**
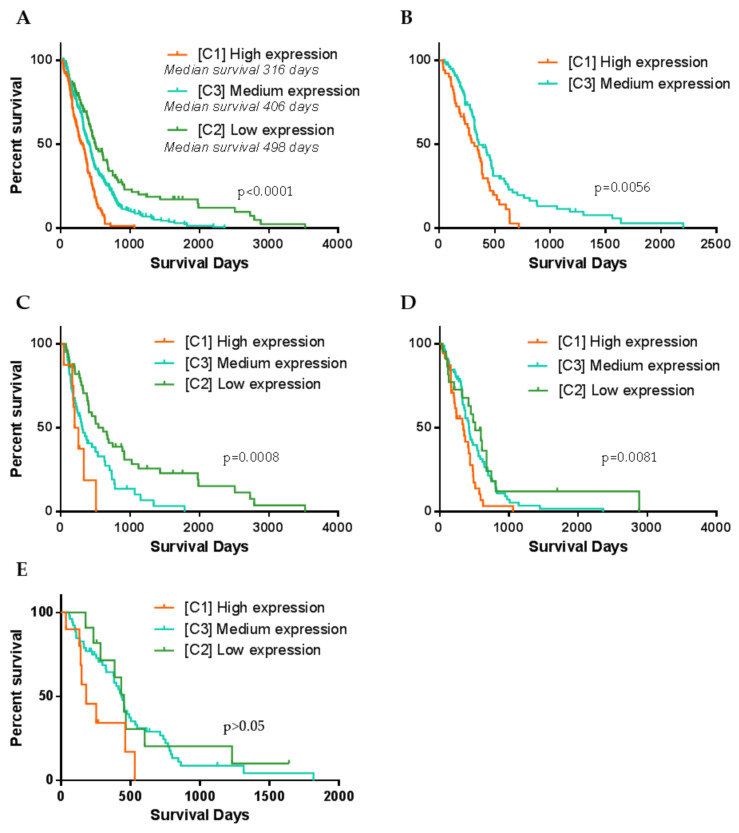
Kaplan–Meier curve plots representing different GBM patients’ groups survival. (**A**) Patient survival curves when specimens were divided according to the cluster dependence. (**B**) Only Mesenchymal GBM subtype patient survival curves calculated according to the cluster dependence. (**C**–**E**) Accordingly, Proneural, Classsical and Neural GBM subtype patient survival curves calculated according to the cluster dependence. *p*-value given based on Log-Rank (Mantel–Cox) test.

Following, we checked if patient clinical features differ between clusters. Survival analysis revealed significant differences in patient’s overall survival between all three clusters, LogRank test, χ^2^ = 42.96, df = 2, *p* < 0.0001, Figure 4A. Kaplan–Meier curves revealed clear separation of survival curves after 120–140 days post-resection. Cluster 1 (high expression) patients have the worst survival prognosis as compared to C2 and C3, median survival was only 316 days (10.4 months). On the contrary, C2 (low expression) patients’ survival prognosis was the most favorable compared to C1 and C3, and patients’ median survival reached 498 days (16.3 months) after tumors resection. Meanwhile, C3 patients showed intermediate survival, median survival was 406 days (13.3 months). Since C1 was consisted of around half of MES subtype specimens and C2 was similarly consisted of around half of PN specimens, such result might be predetermined by subtype itself. Thus, we checked if MES patient survival differ between clusters. The vast majority of MES samples (96%) have fallen in C1 (High) and C3 (medium) clusters (Figure 2B,C), therefore we performed LogRank test for two groups and analysis revealed significantly different distribution of MES patients survival between C1 and C3 clusters, χ^2^ = 7.66, df = 1, *p* = 0.0056, Figure 4B. Survival prognosis of patients belonging to C1 (high expression) was worse than C3 (medium expression) patients. Similarly, we analyzed if PN cases of which the greater part bundled in C2 and C3 clusters differed in patient survival. Analysis revealed a significant association between the distribution of PN patients in clusters and survival, Figure 4C. Similar trends as for the MES subtype were found: PN C1 cluster patients had the worst survival prognosis, C3 cluster patients of PN subtype showed intermediate and C2 showed the most favorable survival prognosis, LogRank test χ^2^ = 14.29, df = 2, *p* < 0.001, Figure 4C. Classical GBM subtype patient survival analysis revealed weaker relation between clusters and survival, nevertheless LogRank test showed significant separation between groups, χ^2^ = 9.64, df = 2, *p* = 0.0081, Figure 4D. From the Kaplan–Meier plot, clear separation can be observed of curves between C1 and the remaining clusters of CL patients survival, nevertheless, there was an obscure difference between C2 and C3, Figure 4D. Neural subtype samples distributed over all three clusters with the largest part in C3, however, survival analysis revealed only the tendencies but not significant differences in patient survival between clusters, LogRank test χ^2^ = 5.41, df = 2, *p* = 0.067, Figure 4E.

### 2.5. Tumor Recurrence in Clusters

**Figure 5:**
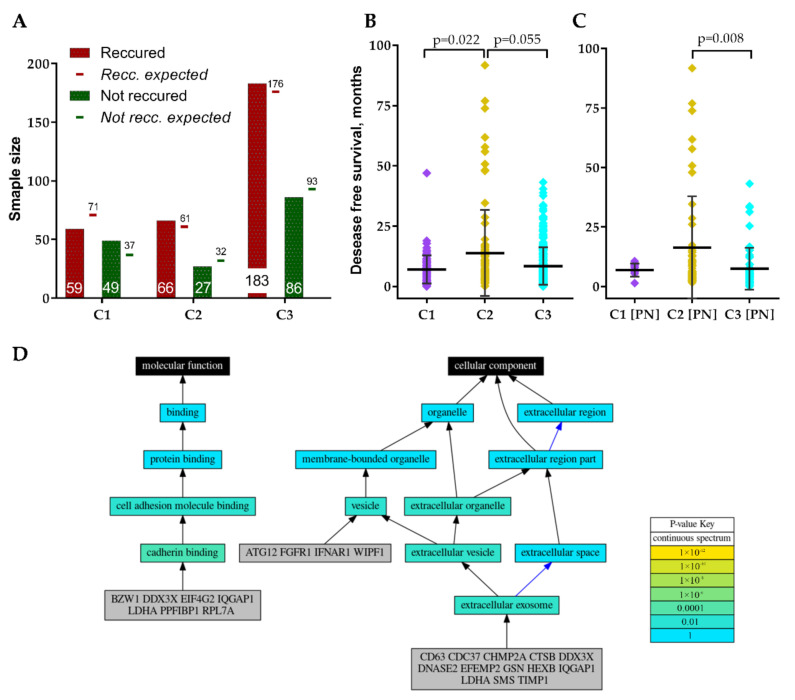
(**A**) Recurred and not recurred samples distribution in clusters. (**B**) GBM patients’ disease-free survival distribution in clusters. (**C**) Proneural subtype patients’ disease-free survival distribution in clusters. (**D**) Schematic visualization of GO analysis results of GSC signature genes.

All three clusters included primary, non-recurrent tumors that recurred or non-recurred until the last follow-up, nevertheless, frequency distribution analysis revealed a significantly different proportion of recurred cases between clusters χ^2^ = 7.64, df = 2, *p* = 0.022, Figure 5A. Cluster 1 revealed the highest disproportion of recurred and not recurred specimens. Comparison of patient disease-free survival between clusters also revealed significant differences, Kruskal–Wallis test *p* = 0.019, Figure 5B. Pairwise comparison showed significantly longer disease-free survival for C2 patients. Comparison of individual GMB subtypes patients’ disease-free survival between clusters revealed that only PN subtype patients had different disease-free survival prognosis between C2 and C3, Kruskal–Wallis test *p* = 0.008, Figure 5C. The remaining subtypes cases disease-free survival did not significantly differ between clusters, nevertheless, MES patients showed a tendency (*p* = 0.155).

### 2.6. GO-Based Functional Annotation of Screened Genes

Since screened GSC specific protein-coding genes were associated with GBM patients’ clinical and pathological data including subtype we decided to perform genes functional annotation. For the analysis, we applied Logically Accelerated Gene Ontology Term Finder (LAGO) tool available online [18]. More than half of selected 30-genes signature genes (*CD63, CDC37, CHMP2A, CTSB, DDX3X, DNASE2, EFEMP2, GSN, HEXB, IQGAP1, LDHA, SMS, TIMP1*) were functionally associated with cellular components related terms, including extracellular exosome [GO:0070062], extracellular vesicle [GO:1903561] and extracellular organelle [GO:0043230] (Bonferroni adjusted *p* < 0.01), Figure 5D. *ATG12, FGFR1, IFNAR1, WIPF1* as well as the genes listed previously were functionally associated with vesicles [GO:0031982]. Seven genes (*BZW1, DDX3X, EIF4G2, IQGAP1, LDHA, PPFIBP1, RPL7A*) were functionally associated with molecular function-related terms, including cadherin binding [GO:0045296] and subsequently cell adhesion molecule binding [GO:0050839] (Bonferroni adjusted *p* < 0.01), Figure 5D. For detailed GO analysis results see Table 2.

## 3. Discussion

In the present study, we screened for glioma stem cells specific gene expression signature by comparing glioblastoma-derived differentiated tumor cells U87-MG and glioblastoma-derived cancer (glioma) stem cells NCH421K. The bulk tumor analysis mainly represents differentiated tumor cells, nevertheless, very important tumor initiating and tumor regenerating cells after treatment—GSC makes up only a small part of the bulk tumor [6,19]. The disease course and patient outcome depend on several main features including patient age, tumor location, tumor molecular composition as well as GSC type and likely amount. However, the main feature responsible for tumor recurrence after gross-total resection is GSCs [19,20,21]. A number of studies were performed to reveal GSC-specific markers, nevertheless, despite several suggested markers [22], the task is still not fully accomplished since GSC and neural stem/progenitor cell (NSC) molecular composition is similar [22]. Moreover, numbers of previous examples demonstrate that single or several markers signature is usually not sufficient for sensitive and specific recognition between similar cells [23]. A multi-gene signature would be a more reasonable solution that would be less sensitive for technical variations arising when preparing a specimen for the analysis, cell cycle predetermined changes and sample heterogeneity decided differences, etc. Thus, current work applied multi-gene signature for the analysis of GBM inter-subtype differences. In the present study, we analyzed if GSC specific signature can be utilized for bulk GBM samples analysis to reveal GBM inter-subtype differences. More than 10 years have passed since GBM were classified into subtypes based on the molecular characteristics [3,16], nevertheless, the significance of subtypes for patient outcomes is still under discussion [4,5,8,24]. It has been shown many times that GBM patient outcome differs depending on the tumor molecular subtype, nevertheless, the magnitude is marginal. Thus, here we used transcriptomic GSC signature assuming that it may represent GSC part in tumors that is responsible for patients’ outcomes. Then we analyzed if patients belonging to the same subtype but having different GSC-specific markers expression levels has different outcomes. Such model revealed that GBM subtypes (MES and PN) which are characterized by stem cells [9,19,25] are showing partitioning of subtypes into different clusters and patients of the same subtype but assigned to different clusters showed significantly different survival and recurrence rates. Previous studies revealed that at least two different types of mesenchymal GSC populations are present within human MES subtype GBM mass [23] what indicates that patient outcomes can also be different depending on the MES GSCs type. The current study also revealed that MES subtype is dividing in two parts and for the first time showed that clinical outcomes between two groups differ significantly. Moreover, we also demonstrated that PN subtype either segregates into two clusters that have significantly different outcomes of GBM patients and different rates of tumor recurrence. Such data indicates that possibly proneural type GSC can also be classified into at least two subpopulations.

The differences of patient outcomes between PN and MES subtypes of GBM [3] as well as differences of cell invasion and angiogenic capacity, aggressiveness, resistance to radiation treatment were shown in vitro and in vivo assays between PN and MES GSC [8,9], nevertheless, inter-subtype variations of both GSC subtypes are not well studied up to date. A high level of GSCs specific genes and worse patient survival prognosis indicates the presence of GSCs in the tumor since GSCs are related to aggressive, invasive, and angiogenic behavior and are resistant to radiation treatment [26].

Gene ontology analysis revealed that the vast majority of screened GSC signature genes were functionally associated with extracellular exosome, and vesicles terms. Extracellular vesicles as a distinctive feature of GSC were described in a few publications to date [27], revealing its applicability as a GSC specific biomarker. Current findings are in the line with recently published data where extracellular vesicles (EV) were found to have very important role as biological mediators in GBM. Moreover, EV were found to be specific to molecular subtype and functional state of donor cancer cells, including cancer stem cells. [10]. Spinelli et al. showed that PN and MES GSC lines produce vastly different populations of EVs, including varying EV profiles, marker distributions and proteomes [10], and this indicates that molecular processes involved in EV biogenesis pathways in GSC MES and PN subpopulations of subtypes might be employed as a GSC subpopulation specific biomarkers.

The current comparison between GBM tumor and glioma stem cell lines involved only a single type of each cell line and that should be stated as the limitation of the study. Based on the current knowledge, tumor cells and tumor stem cells are driven by different biological pathways that in turn results in distinct phenotypic states, functional attributes, and different principal properties between cell types [28]. Despite the implication that different lines of glioma stem cells are more similar to each other as compared to differentiated cancer cells, the usage of several lines would also lead to assess variations between cells of the same type. Moreover, the present study included NCH421K glioma stem cell line that was described as having mesenchymal subtype properties [29], thus the application of proneural GSC would likely reveal a more complete picture. Nevertheless, the proposed signature revealed clinically relevant segregation not only of mesenchymal subtype GBM patients but also of proneural subtype. In conclusion, our study revealed that glioblastoma CSCs specific genes could be applied to supplement glioblastoma subtype information. Current findings also revealed branching of molecular GBM subtypes that were significantly linked to patient outcome and that might be decided by distinct GSC subpopulations.

## 4. Materials and Methods

### 4.1. Cell Lines and Culturing

Human glioblastoma derived GBM stem-like cells NCH421K and human glioblastoma cell line U87-MG were used for the screening part. NCH421K cell line were kindly provided by PI Aiste Jekabsone from the Institute of Pharmaceutical Technologies at Lithuanian University of Health Sciences (Kaunas, Lithuania) and were cultivated in serum-free medium consisted of: Dulbecco’s Modified Eagle MediumNutrient Mixture F-12 Ham (Sigma-Aldrich, cat. no. D8437, Merck KGaA, Steinheim, Germany) containing 0.8 g/L Glucose (Gibco, cat. no. A2494001, Life Technologies, Cramlington, UK), 1 × MEM Non-Essential Amino Acids Solution (Gibco, cat. no. 11140035, Life Technologies, Cramlington, UK), 1 × Penicillin-Streptomycin (Gibco, cat. no. 15140122, Life Technologies, Grand Island, NY, USA), 0.012% Bovine Albumin Fraction V (7.5% solution) (Gibco, cat. no. 15260037, Life Technologies, Cramlington, UK), 0.5 × B-27™ Supplement, serum free (Gibco, cat. no. 17504044, Life Technologies, Grand Island, NY, USA), 0.5 × N-2 Supplement (Gibco, cat. no. 17502048, Life Technologies, Grand Island, NY, USA), 0.05 mM 2-Mercaptoethanol (Gibco, cat. no. 31350010, Life Technologies, Bleiswijk, The Netherlands). Fibroblast growth factor 20 ng/mL (Chemicon, cat. no. GF003, Merck KGaA, Gernsheim, Germany) and epidermal growth factor 20 ng/mL (Chemicon, cat. no. GF144, Merck KGaA, Gernsheim, Germany) supplements were freshly added each time before splitting cells. Cells were grown in ordinary flasks as spheroids and harvested by centrifuging, discarding media and stored at −80 °C.

U87-MG cells were cultivated in DMEM, high glucose, GlutaMAX supplement medium (Gibco, cat. no. 61965059, Life Technologies, Cramlington, UK), supplemented with 10% of heat inactivated FBS (Gibco, cat. no. 10500064, Life Technologies, Darmstadt, Germany (Brazil origin)) and 1% of Penicillin-Streptomycin solution (Gibco, cat. no. 15140122, Life Technologies, Bleiswijk, The Netherlands). When the confluence reached ~75% cells were scraped off the culture vessel surface in cold D-PBS using rubber tipped cell scrapers, centrifuged, and stored at −80 °C.

### 4.2. RNA Purification, mRNA Enrichment and Sequencing

RNA from cell pellets was purified using TRIzol reagent (Ambion, cat. no. 15596026, Life Technologies, Carlsbad, CA, USA). Dynabeads™ mRNA DIRECT™ Purification kit (Invitrogen, cat. no. 61012, ThermoFisher Scientific, Vilnius, Lithuania) was used for polyadenylated RNA enrichment. The quality and quantity of isolated RNA was controlled using NanoDrop, Agilent Bioanalyzer (RNA 6000 Nano Kit, Agilent, cat. no. 5067–1511, Vilnius, Lithuania), and agarose-gel electrophoresis. Direct RNA sequencing kit (Oxford Nanopore, cat. no. SQK-RNA002, Oxford, UK) was used to prepare 3′-polyA RNA for sequencing. 500 ng of prepared RNA was used for sequencing. RNA-seq analysis was performed on MinION Oxford Nanopore sequencing system using single cell per sample and analyzed with “MasterOfPores” pipeline consisting of pre-process and poly-A length estimation (NanoTail) modules.

### 4.3. TCGA Gene Expression Data Processing

The Cancer Genome Atlas (TCGA) coordination center data [14] was utilized for in silico analysis of signature relevance for GBM subtypes in human specimens. TCGA gene expression data (received by gene expression array platform Affymetrix HT Human Genome U133a; *n* = 539) of GBM patients with known survival data, tumor recurrence information, and TCGA (Verhaak et al., 2010) subtype were collected from UCSC Xena [30] database. Four main criteria were applied to select proper cases for the analysis: only primary non-recurrent tumors cases; only patients who did not receive treatment prior to resection, only patient who survived more than 30 days after resection and only cases with known GBM subtype information were selected. Both: *IDH1* wild-type and mutant cases were used for the study. After the data filtering 468 GBM cases remained out of Expression data were normalized within each feature (gene) applying standard score: z = (x − µ)/σ.

### 4.4. Statistical Analysis

Differences across three or more independent groups were analyzed applying the Kruskal–Wallis test. Chi-square test used for categorical data analysis. Survival analysis was done applying Kaplan–Meier curve method, and log-rank test used to compare difference of survival curve across groups. To show the reliability of the survival estimate, the confidence interval (CI) with 95% confidence level was presented. Orange Data Mining (V3.28, University of Ljubljana) machine learning and data visualization soft as well as GraphPad Prism (V6.01, Graph-Pad Software, Inc., San Diego, CA, USA) software were used for data analysis. The level of significance was *p* < 0.05.

## Figures and Tables

**Table 1:** Summarized table of patients demographical, clinical and specimen’s molecular data distribution between clusters.

Features	Cluster			*p*-Value
	C1 (*n* = 106)	C2 (*n* = 93)	C3 (*n* = 269)	
Gender *n* = 468 (%)				
Female	39 (36.8%)	41 (44.1%)	102 (37.7%)	*p* = 0.477 ^#^
Male	67 (63.2%)	52 (55.9%)	167 (62.3%)	
Age, years (median) [mean]	60.7 [60.5]	54.6 [54.3]	60 [58.7]	*p* = 0.038 ^§^
Survival, months				
(median) [mean]	10.4 [10.1]	13.3 [21.6]	16.3 [14.5]	*p* < 0.001 *
GBM Subtype *n* = 468 (%)				
Mesenchymal	52 (49.1%)	7 (7.6%)	79 (29.3%)	*p* < 0.001 ^#^
Proneural	8 (58%)	54 (55.9%)	60 (22.4%)	
Classical	35 (33%)	22 (23.7%)	78 (29%)	
Neural	11 (10.4%)	10 (10.8%)	52 (19.3%)	
*IDH1* status *n* = 355 (%)				
Wild-type	90 (100%)	51 (75%)	188 (95.4%)	*p* < 0.001 ^#^
Mutant	0 (0%)	17 (25%)	9 (4.6%)	
*MGMT* status *n* = 304 (%)				
Unmetylated	49 (54.4%)	19 (41.3%)	83 (49.4%)	*p* = 0.348 ^#^
Methylated	41 (45.6%)	27 (58.7%)	85 (50.6%)	
G-CIMP *n* = 459 (%)				
G-CIMP	0 (0%)	26 (28.9%)	11 (4.2%)	*p* < 0.001 ^#^
non-G-CIMP	106 (100%)	64 (71.1%)	254 (95.8%)	

#—*p*-value estimated by Pearson Chi-square (χ^2^) test, §—*p*-value estimated Kruskal–Wallis test, *—*p*-value estimated by Log-rank test.

**Table 2:** Gene ontology (GO) analysis results of screened GSC specific 30-gene signature genes.

GO ID	Term	*p*-Value	Uncorrected *p*-Value	Number Annotated	Annotated Genes
GO:0045296	cadherin binding	0.000365	4.31 × 10^−7^	7	*BZW1, DDX3X, EIF4G2, IQGAP1, LDHA, PPFIBP1, RPL7A*
GO:0070062	extracellular exosome	0.00372	4.40 × 10^−6^	13	*CD63, CDC37, CHMP2A, CTSB, DDX3X, DNASE2, EFEMP2, GSN, HEXB, IQGAP1, LDHA, SMS, TIMP1*
GO:1903561	extracellular vesicle	0.00416	4.92 × 10^−6^	13	*CD63, CDC37, CHMP2A, CTSB, DDX3X, DNASE2, EFEMP2, GSN, HEXB, IQGAP1, LDHA, SMS, TIMP1*
GO:0043230	extracellular organelle	0.00420	4.97 × 10^−6^	13	*CD63, CDC37, CHMP2A, CTSB, DDX3X, DNASE2, EFEMP2, GSN, HEXB, IQGAP1, LDHA, SMS, TIMP1*
GO:0050839	cell adhesion molecule binding	0.00726	8.59 × 10^−6^	7	*BZW1, DDX3X, EIF4G2, IQGAP1, LDHA, PPFIBP1, RPL7A*
GO:0031982	vesicle	0.00758	8.97 × 10^−6^	17	*ATG12, CD63, CDC37, CHMP2A, CTSB, DDX3X, DNASE2, EFEMP2, FGFR1, GSN, HEXB, IFNAR1, IQGAP1, LDHA, SMS, TIMP1, WIPF1*

## Data Availability

The Cancer Genome Atlas (TCGA) Research Network datasets were utilized in the study. Data available from: https://xenabrowser.net/ (accessed on 5 May 2021).
